# Use of EN Snare Device for Successful Retrieval of a Pigtail Catheter Straightener after Iliac Artery Stent Placement: A Case Report

**DOI:** 10.3400/avd.cr.26-00068

**Published:** 2026-09-15

**Authors:** Takeshi Wada, Katsutoshi Takayama, Kaoru Myouchin

**Affiliations:** Department of Interventional Neuroradiology/Radiology, Kouseikai Takai Hospital, Tenri, Nara, Japan

**Keywords:** EN Snare, percutaneous retrieval, pigtail catheter straightener

## Abstract

Percutaneous retrieval is a well-established, minimally invasive method for removing foreign bodies from blood vessels. We experienced a 77-year-old man in whom a radiolucent pigtail catheter straightener had been mis-inserted into the abdominal aorta and was successfully retrieved using an EN Snare device. This report demonstrates that operators must remain aware of the risk of accidentally inserting pigtail catheter straighteners into blood vessels.

## Introduction

Percutaneous removal of intravascular foreign bodies (IFBs) is a less invasive technique than surgical removal. Forceps, baskets, and snare devices have been used for this purpose. IFBs are most often caused iatrogenically and can include wire pieces, catheter pieces, inferior vena cava filters, coils, and stents. We report a rare case in which stent placement was performed for stenosis of the common femoral artery using a transradial approach, where a pigtail catheter straightener was found to have been mis-inserted into the lower end of the abdominal aorta during treatment 46 d earlier. The straightener was found and successfully retrieved using an EN Snare device. The patient provided consent for the publication of this report.

## Case Report

**Fig. 1:**
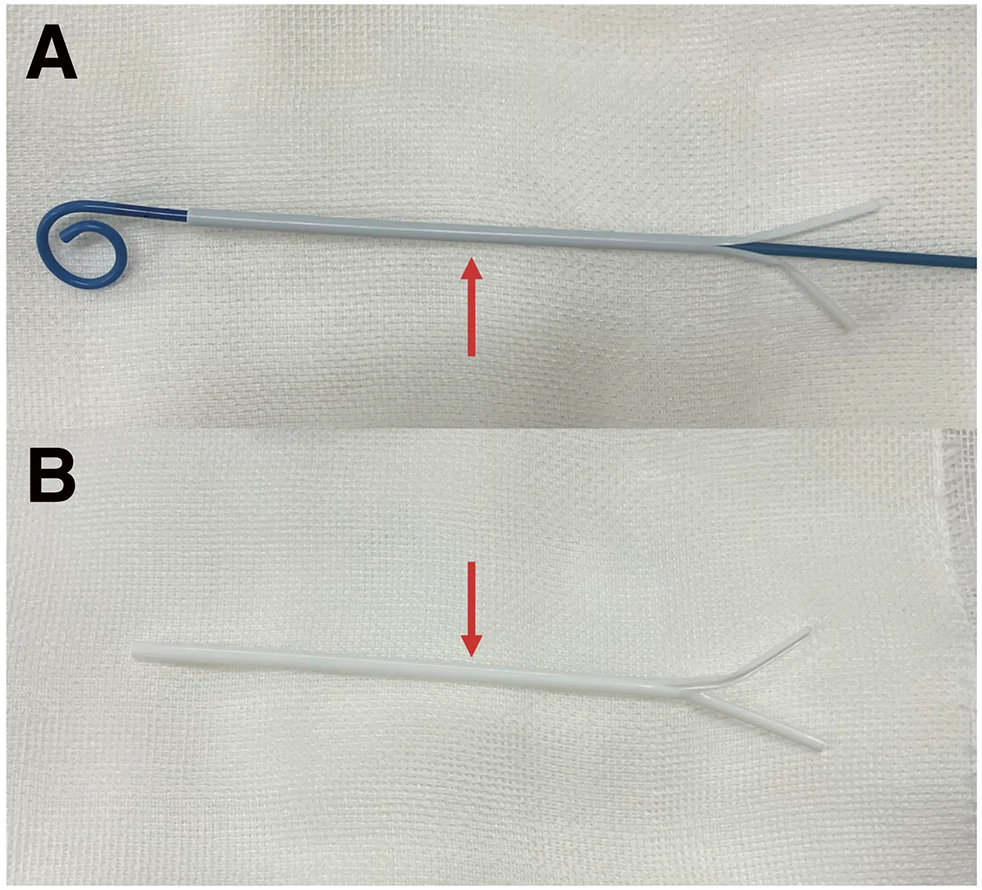
Digital photos. (**A**) The pigtail catheter shown along with its straightener (arrow). (**B**) The straightener alone (arrow).

The patient was a 77-year-old man who presented with the chief complaint of left intermittent claudication. Initial computed tomographic angiography (CTA) revealed stenosis due to a short-segment dissection of the left common iliac artery as the cause of claudication. He underwent endovascular stent placement via transradial artery access using a 6-Fr guiding sheath and a self-expanding stent (BRITE TIP RADIANZ and S.M.A.R.T. RADIANZ; Cordis, Miami Lakes, FL, USA). Final aortography using a 4-Fr pigtail catheter (Trail HF; FUKUDA DENSHI, Tokyo, Japan) (**Fig. 1**) demonstrated correct positioning of the stent successfully. After the procedure, the loss of the pigtail catheter straightener (8.5 cm in length and 2 mm in diameter) was not noticed, and no foreign body was identified on live X-ray images or digital subtraction angiography (DSA). The patient was discharged without any complications.

Forty-six days after that procedure, the presence of a tubular foreign body in the lower abdominal aorta was suspected on plain computed tomography (CT) of the abdomen performed to investigate right lower abdominal pain. At that time, we realized the foreign body was the mis-inserted pigtail catheter straightener.

**Fig. 2:**
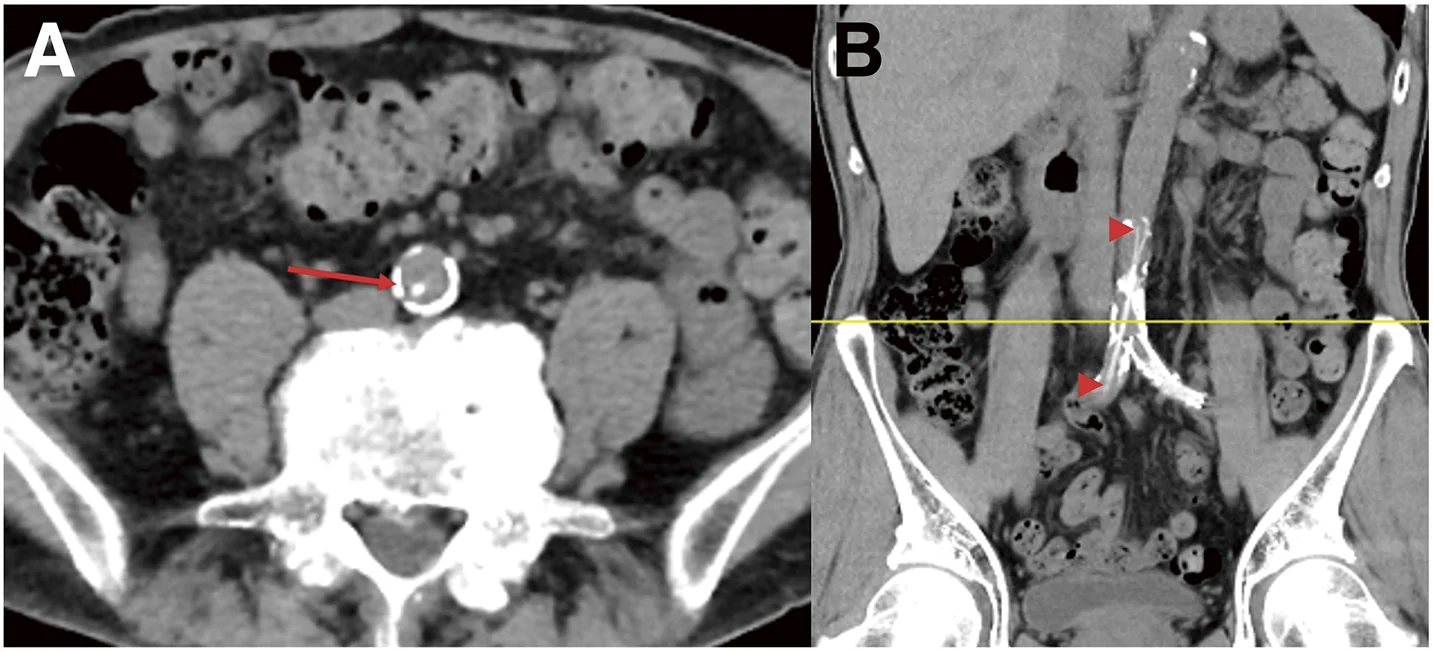
Plain computed tomography of the abdomen. (**A**) Axial image demonstrating a foreign body consistent with a pigtail catheter straightener (arrow). (**B**) Coronal image confirming the position of the foreign body between the lower abdominal aorta and the right common iliac artery (arrowheads).

**Fig. 3:**
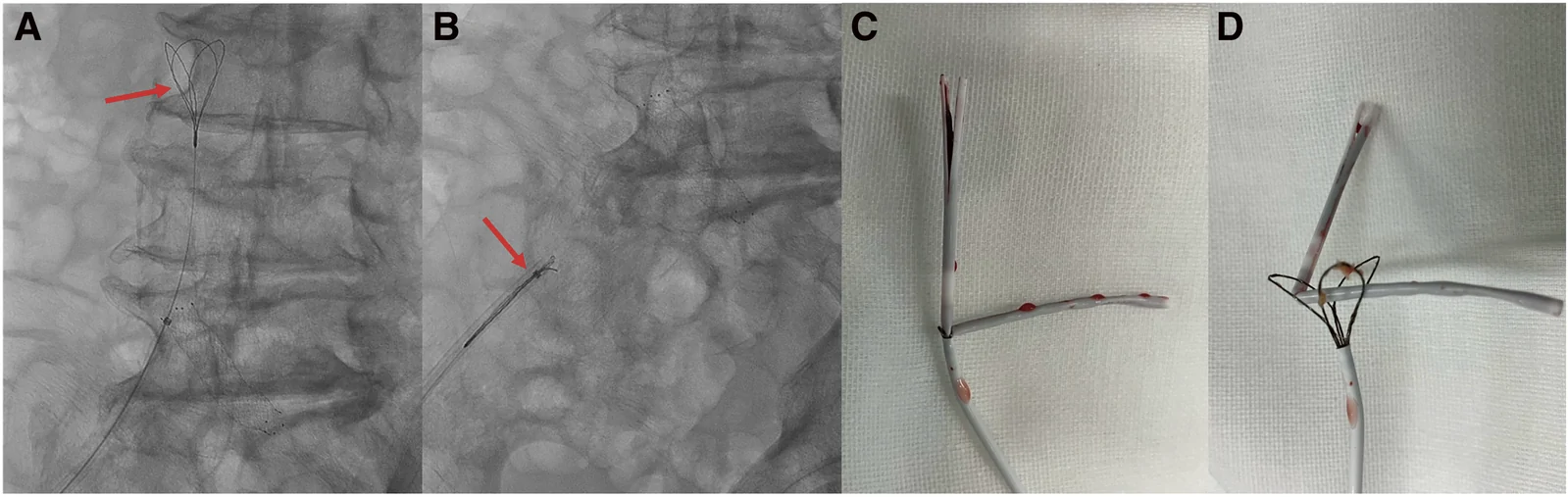
Abdominal radiographs and digital photos obtained during retrieval of the catheter straightener. (**A**) Radiograph showing the deployed EN Snare (arrow) at the level of the 3rd lumbar vertebra. (**B**) Pulling back of the EN Snare into the right femoral sheath (arrow). (**C**) Digital photo showing the retrieved catheter straightener and EN Snare. (**D**) Retrieved catheter straightener and an artificially partially deployed EN Snare.

Percutaneous retrieval of the foreign body was attempted 48 d after stent placement. Although we retrospectively checked the abdominal radiographs from the time of the previous operation, no evidence of radiopaque foreign bodies was noted in the abdomen. Preoperative CT was therefore used to confirm the position of the head and tail of the foreign body, which demonstrated the presence of a tubular foreign body consistent with a pigtail catheter straightener between the lower abdominal aorta and the right common iliac artery (**Fig. 2A** and **2B**). Subsequently, a triple-loop snare device (EN Snare 6-Fr, 15–20 mm; Merit Medical, Sough Jordan, UT, USA) for percutaneous retrieval of foreign bodies was introduced by means of a right common femoral artery puncture (25 cm and 9-Fr angiographic sheath; Medikit, Tokyo, Japan). The EN Snare was deployed at the level of the 3rd lumbar vertebra, and the retraction and grasping were performed several times to grasp it, and the loop was closed around the foreign body (there was incomplete closing of the loop around the foreign body). We pulled back the EN Snare and the foreign body into the right femoral sheath, and the foreign body and EN Snare were then removed (**Fig. 3A**–**3D**). Post-angiography was performed after retrieval, and no vascular injury, thrombus, or embolic complication was observed. Cone-beam CT (CBCT) also showed no evidence of foreign bodies.

## Discussion

Most IFBs are radiopaque (catheter or guidewire fragments, vena cava filters, embolic coils, endovascular stents) to facilitate precise positioning and use under fluoroscopy. When necessary, radiopaque foreign bodies can be removed percutaneously using fluoroscopy.^1–6)^ In contrast, radiolucent foreign bodies are more difficult to detect and may remain unrecognized during the procedure. In the present case, the pigtail catheter straightener was completely radiolucent and could not be identified on fluoroscopy or DSA, resulting in delayed recognition 46 d after the initial intervention. This highlights the importance of considering radiolucency when using catheter accessories and the need for alternative imaging modalities such as CT for accurate localization.

**Fig. 4:**
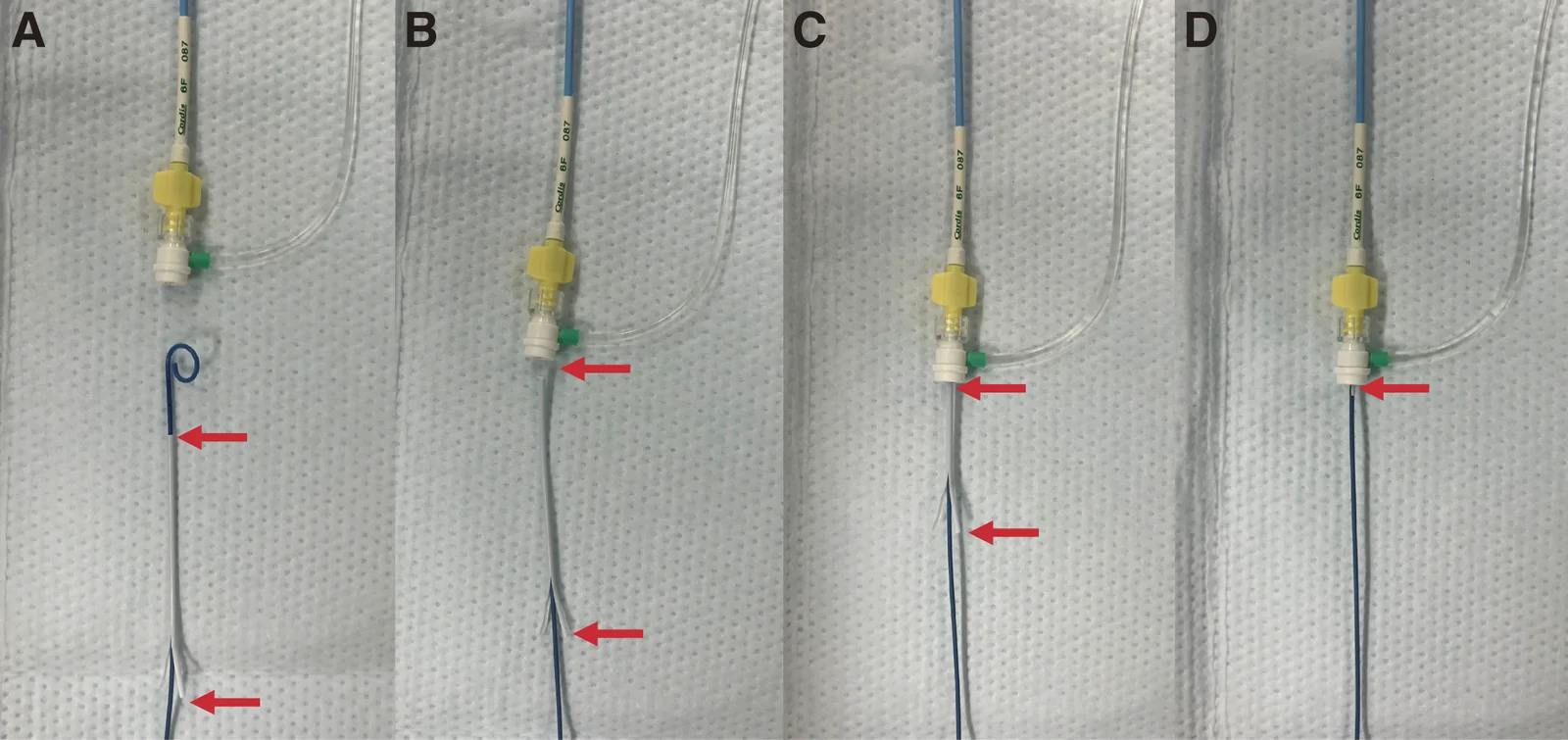
In vitro insertion of a 4-Fr pigtail catheter into a 6-Fr guiding sheath. (**A**) A 4-Fr pigtail catheter with its straightener (arrows) before insertion into a 6-Fr guiding sheath (**B**, **C**) The pigtail catheter and its straightener (arrows) during advancement into the sheath. (**D**) The 4-Fr pigtail catheter straightener is nearly fully inserted through the 6-Fr guiding sheath without resistance (arrow).

The mechanism of accidental mis-insertion warrants careful consideration. The straightener used in this case was a straight peel-away type without a protruding stopper, and the 4-Fr pigtail catheter was inserted through a 6-Fr guiding sheath. This size mismatch likely allowed the straightener to pass through the sheath valve without resistance. Furthermore, the transradial approach may reduce tactile feedback compared with transfemoral manipulation, making it more difficult to perceive subtle resistance (**Fig. 4**). These factors likely contributed to the unnoticed intravascular insertion. Retrospective review of the final angiogram revealed no filling defect, consistent with the radiolucent nature of the device.

Accurate pre-procedural localization was essential for successful retrieval. CT clearly demonstrated the position and orientation of the straightener, enabling precise planning of the retrieval strategy. The EN Snare was selected because its triple-loop configuration provides a wide capture range and multidirectional engagement, which is advantageous when grasping a smooth, radiolucent tubular object. Tactile resistance was felt before the foreign body was captured by the EN Snare, confirming secure engagement even though the loop could not be fully closed. Although other devices such as gooseneck snares or microsnares may also be effective, the EN Snare offered practical advantages in this setting.

The foreign body remained asymptomatic for 46 d. This may be explained by the straightener’s flexibility, smooth surface, and small diameter, which likely minimize endothelial irritation and thrombus formation. Its longitudinal orientation along the aortic flow axis may also have reduced hemodynamic disturbance. The straightener was incidentally detected on plain CT. Although the patient presented with right lower abdominal pain, there was no change in symptoms after the foreign body was removed. Therefore, this symptom was not considered related to the foreign body.

Two previous reports have described retrieval of pigtail catheter straighteners.^7,8)^ In both cases, multimodal imaging—including CT, DSA, ultrasound, or CBCT—was essential for localization. Our experience similarly demonstrates that CT is indispensable when dealing with radiolucent foreign bodies. In the report by Barbiero et al.,^7)^ a mis-inserted catheter straightener of a 5-Fr pigtail catheter could not be detected on fluoroscopic images but was incidentally detected on contrast-enhanced CT 5 d after endovascular stent-graft deployment for an abdominal aortic aneurysm. They used DSA and ultrasound to locate the catheter straightener, which was retrieved 39 d after the procedure using a gooseneck snare. Izumi et al.^8)^ reported retrieval of a pigtail catheter straightener during infrarenal aortic stent placement. The foreign body mis-inserted into the aorta was detected by plain X-ray and CBCT. The use of CBCT can facilitate quick and safe retrieval. The foreign body was successfully retrieved by the lateral grasp technique using a microsnare and microwire.

To prevent the pigtail catheter straightener from being accidentally inserted into the vessel through the sheath safety valve, the typical pigtail catheter straightener is angled and has a small protrusion. In the present case, however, the pigtail catheter straightener was a straight peel-away type with no protrusion (**Fig. 1**), and the 4-Fr pigtail catheter was inserted into a 6-Fr guiding sheath. This likely led to mis-insertion into the body without noticing the resistance. The foreign body was not identifiable on X-ray images. After confirming the location of the foreign body on CT, removal was achieved using the EN Snare. When inserting a pigtail catheter inside a thick sheath, care must be taken to always be aware of this possibility.

Preventive measures are critical. Because straighteners may be radiolucent and difficult to detect fluoroscopically, operators should confirm the presence and integrity of the straightener after catheter insertion and removal. Implementing a procedural checklist similar to surgical gauze or needle counts may help prevent delayed recognition of missing catheter accessories. When inserting a pigtail catheter through a large-bore sheath, particular attention should be paid to the possibility of inadvertent straightener migration.

## Conclusion

This report demonstrates that operators must use caution to avoid accidentally inserting a pigtail catheter straightener into blood vessels, although we successfully achieved percutaneous retrieval of a radiolucent pigtail catheter straightener using an EN Snare system.

## Declarations

### Data availability

The data that supports the findings of this study are available from the corresponding author, Takeshi Wada, upon reasonable request.
